# Prevalence of stress associated oral mucosal disorders among patients from Southern Rajasthan, India

**DOI:** 10.6026/973206300200754

**Published:** 2024-07-31

**Authors:** Srujal Jivrajani, Kavita Verma, Saurabh Goel, Abhishek Jayaswal, Rinky Kukreja, Mili Dube

**Affiliations:** 1 Department of Oral Medicine and Radiology, Pacific Dental College and Research Center, Udaipur, Rajasthan, India; 2 Department of Pedodontics and Preventive Dentistry, Pacific Dental College and Research Centre, Udaipur, Rajasthan, India

**Keywords:** Stress associated oral mucosal lesions/conditions, apthous ulcers, burning mouth syndrome, myofacial pain dysfunction syndrome

## Abstract

Data on the prevalence of stress associated oral mucosal lesions/conditions such as oral lichen planus, apthous ulcers, burning mouth syndrome, headache, xerostomia, halitosis, myofacial pain dysfunction syndrome amongst population of Southern Rajasthan is of interest to dentists. Cross sectional study had been conducted on 5214 patients from 18-60 years age group who visited the Department of the Oral Medicine and Radiology, Pacific Dental College and Research Centre, Bedla, Udaipur. Findings such as burning of oral mucosa, presence of vesicles and ulcers, striation in oral cavity, limitation of jaw movement, muscular pain were assessed for the establishment of diagnosis. prevalence of stress associated oral mucosal lesions/conditions like oral lichen planus, apthous ulcers, burning mouth syndrome, headache, xerostomia, halitosis, myofacial pain dysfunction syndrome was reported to be 12%, 17%, 3%, 21%, 6 %, 23 and 18% respectively. Besides stress management therapy, Professional consultation along with proper investigation and medicinal treatment is required.

## Background:

Stress was originally derived from the Latin word "stringi" which means, "to be drawn tight." Stress is a psycho physiological response of the organism to a perceived challenge or threat as defined by Brevik *et al.* in the year 1996. Stress is not what happens to someone, but how someone reacts to what happens [1]. It can also be defined as adoptive response of an organism to a threatening stimulus, which provides the link between the psychological and physiological processes that are associated in the onset of disease [2]. Both physical and psychological stress affect different aspects of the immune system, partly by increasing serum catecholamines and corticosteroids [3] and as a side effect to prolonged exposure to these chemical changes body develops endocrinal, metabolic homeostatic immunological disturbances leading to diseases such as hypertension, diabetes mellitus, cardiovascular disease, periodontal emotional stress syndrome, osteoporosis, rheumatoid arthritis, inflammatory bowel disease, preterm delivery are related stress either as a physiological response to stress or as a behavioral response [4]. The oral cavity represents an organ of the expression as it is connected specifically or emblematically to the significant human senses and interests and is charged with a high psychologic potential. Certain diseases which affect the oral mucosa may be the direct or indirect expression of emotions or conflicts [5, 6]. Hence, Stress has a direct relation with oral lesions and conditions (directly proportional), *i.e.*, there are many studies which state that, oral lesions become more prominent when the level of stress rises above the threshold. For example Lichen Planus, Recurrent apthous stomatitis, where severity of oral lesions increases along with increase in level of stress [7, 8]. Therefore, it is of interest to determine the prevalence of stress associated oral mucosal lesions/conditions such as Oral lichen planus, Apthous ulcers, Burning mouth syndrome, Headache, Xerostomia, Halitosis, Myofacial pain Dysfunction syndrome amongst population of Southern Rajasthan.

## Materials and Methods:

A cross sectional study had been conducted on 5214 patients from 18-60 years age group who visited the Department of the Oral Medicine and Radiology, Pacific Dental College and Research Centre, Bedla, Udaipur after obtaining ethical clearance from the ethical committee. An informed consent had been taken for all the patients. They were subjected to thorough case history related to stress and a detailed clinical examination of the oral cavity was carried out under artificial light. On intra oral examination findings such as burning of oral mucosa, presence of vesicles and ulcers, striation in oral cavity, limitation of jaw movement, muscular pain and some routine parameters included in the establishment of diagnosis for the conditions such as Oral lichen planus, Apthous ulcers, Burning mouth syndrome, Headache, Xerostomia, Halitosis, Myofacial pain Dysfunction syndrome was done according to diagnostic criteria [9]. Patient having habit history of tobacco, areca nut or alcohol, having drug history, any graft placement or restoration, taking any treatment for oral lesions or suffering from carcinoma had been excluded from the study Following establishment of diagnosis, each patient was informed about the condition, its precancerous potential.

## Results and Discussion:

The data shows that out of 5214 cases; the prevalence of stress associated oral mucosal lesions/conditions was reported to be 25.4 % being observed in 1325 cases. Amongst them 38% were reported to be males and 62% were females. Hence, female predominance in association with occurrence of stress associated oral mucosal lesions is seen. This could be explained that females are more sensitive to stress and emotional disturbances which could affect their immunity in turn a leading cause of occurrence of oral mucosal lesions as reported by Chaturvedi *et al.* [10]. Maximum number of patients diagnosed with stress associated oral mucosal lesions had been observed to be among young age group of 20-25 years. The least number of patients had been observed in the age group exceeding 50 years. Thus, younger age group are prone to stress associated oral mucosal lesions.

Prevalence of stress associated oral mucosal lesions/conditions like oral lichen planus, apthous ulcers, burning mouth syndrome, headache, xerostomia, halitosis, myofacial pain dysfunction syndrome was reported to be 12%, 17%, 3%, 21%, 6%, 23% and 18% respectively. This is because psychological stress affects the normal nerve-endocrine-immune pathway of the human body, leading to over-activation of the hypothalamus-pituitary-adrenal (HPA) axis and sympathetic nerve-adrenal medulla. The hypothalamus-pituitary-gonad (HPG) axis is regulated by the HPA axis, which can inhibit or over activate endocrine and immune systems, and the secretion of corresponding hormones, cytokines and proteins changes accordingly This may damage the oral mucosa and induce disorders such as lichen planus, apthous ulcers and others [11].

## Conclusion:

The prevalence of stress associated oral mucosal lesions had been reported in 25.4% of the population. Moreover, significant association of young age group (20-25) years among female had been observed. However, further studies should be conducted with a large sample size to determine the role of stress in occurrence of oral mucosal lesions.

## Financial support:

NIL
